# Cutaneous leiomyosarcoma – Case report

**Published:** 2014-06-25

**Authors:** ME Ciurea, CV Georgescu, CC Radu, CC Georgescu, LE Stoica

**Affiliations:** * Department of Plastic and Reconstructive Surgery, Craiova University of Medicine and Pharmacy; ** Department of Histopathology, County Emergency Hospital, Craiova; *** Department of Dermatology, Craiova University of Medicine and Pharmacy; **** Department of Pharmacology, Craiova University of Medicine and Pharmacy; Department of Anesthesiology and Intensive Care, County Emergency Hospital, Craiova

**Keywords:** leiomyosarcoma, skin, pleomorphic

## Abstract

Abstract

Cutaneous leiomyosarcoma (CLM) is a very rare smooth muscle tumor arising from the dermis or subcutaneous tissue in the skin. Superficial leiomyosarcoma originates in the superficial dermis or subcutaneous tissue and represents about 3% of the soft tissue sarcomas. CLM presents in persons of all ages but with a peak between 60-70 years old. It may also occur anywhere on the body; the existing cases reported it on the face and trunk. The clinic of leiomyosarcoma consists in a firm dermal nodule, which can be painful, pruritic or paresthestic. The tumor is of 1-3 cm in diameter and can often be seen as a solitary formation. We report one case of a cutaneous leiomyosarcoma arising in the chest region of a 79- year-old male. Leiomyosarcoma is a rare entity whose clinical presentation may appear nonspecific, making diagnosis difficult. Primary tumor excision with wide oncological safety margins is considered, when suitable case, the most appropriate method. Other therapeutic methods, such as radio- or chemotherapy are described as without significant benefits. Despite the claims of radical surgical treatment, due to recurrence rates, the prognosis remains poor. We recommend long-term follow-up of patients to capture a subsequent malignant disease progression.

## Introduction

Cutaneous leiomyosarcoma (CLM) is a very rare smooth muscle tumor arising from the dermis or subcutaneous tissue in the skin. The incidence is of less than 3% of the cutaneous soft-tissue sarcomas.

 Generally, superficial leiomyosarcomas occur as a solitary, slowly growing lesion most commonly on proximal extremities, which coincide with the hair bearing areas. It may also occur anywhere on the body, the existing cases being reported on the face and trunk. Only 10-15% of the subcutaneous leiomyosarcomas arise in the trunk [**1,2**]. CLM presents in persons of all ages but with a peak between 60-70 years old. Preoperative misdiagnosis is common because it is a rare malignant tumor.

 The etiology of these tumors is relatively unknown, although ionizing irradiation, sunlight, antecedent traumatic injury, chemicals and lupus vulgaris have been associated with this type of tumor [**3**].

 Superficial leiomyosarcomas have two subdivisions, cutaneous and subcutaneous forms due to their different original, clinical and prognostic implications [**4**]. Subcutaneous tumors have been reported to be associated with a higher risk of local recurrences and distant metastases, compared to their cutaneous counterparts. 

## Case report

**Fig. 1:**
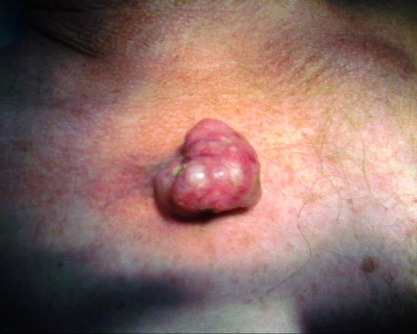
Clinical aspect: well-demarcated, irregular, firm erythematous nodule on chest region

 A 79-year-old male presented to our department with a painless nodule on chest region, which had been progressively increasing in size for 10 years. Physical examination revealed an irregular, firm, tender exophytic swelling, measuring 4 cm diameter at the anterior trunk (**Fig. 1**). There were no signs of infection and the overlying skin was normal. The remainder of the clinical examination was normal. Lymph nodes were not palpable. His past history was significant of a local trauma. 

 Systemic investigations, including complete blood count, fasting blood sugar level, liver function test and renal function test, were within normal limits. Hepatitis B virus surface antigen and human immunodeficiency virus enzyme-linked immunosorbent assay were non-reactive. 

 A clinical diagnosis of dermatofibrosarcoma was considered. 

 A surgical resection with wide margins of at least 2 cm was performed. 

**Fig. 2:**
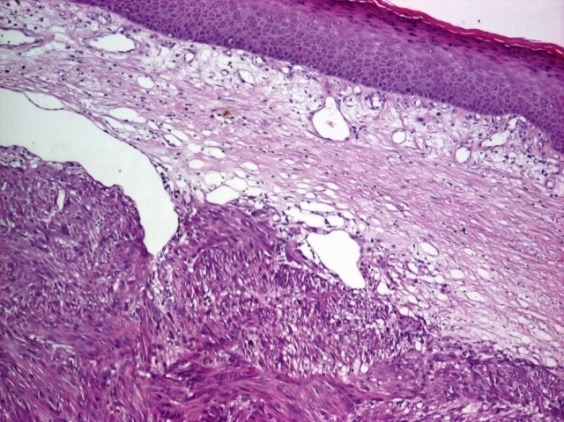
Histopathological features of cutaneous leiomyosarcoma: hypodermis invasion, elongated cells arranged in intersecting bundles, pleomorphic nuclei (HE stain x 40)

 Histopathological examination revealed a tumor consisting of elongated malignant cells characterized by nuclear polymorphism combined with atypical mitoses. It also revealed the tumor extension in the hypodermis, showing a fasciculated growth pattern of spindle cells with hyperchromatic and pleomorphic nuclei and eosinophilic cytoplasm (**Fig. 2**). 

**Fig. 3:**
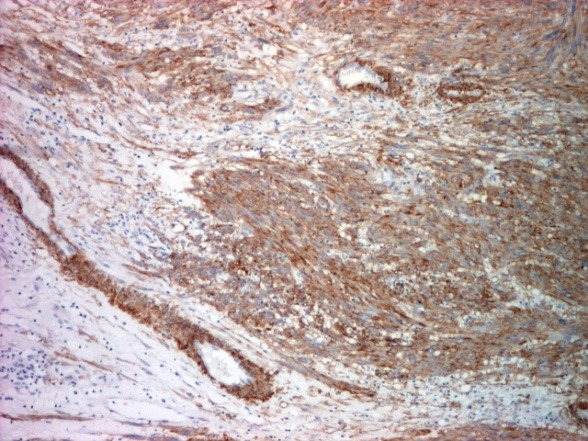
Immunohistochemical stain for α smooth muscle actin: diffuse positive in tumor cells and large vessel walls (x 40)

 The surgical margins were free of disease. On a detailed immunohistochemical analysis, the tumor cells were intensely positive for smooth muscle actin (SMA), moderately positive for vimentin (**Fig. 3^4^**) and negative for CD34 that was positive only in the vessels endothelium (**Fig. 5**). 

**Fig. 4:**
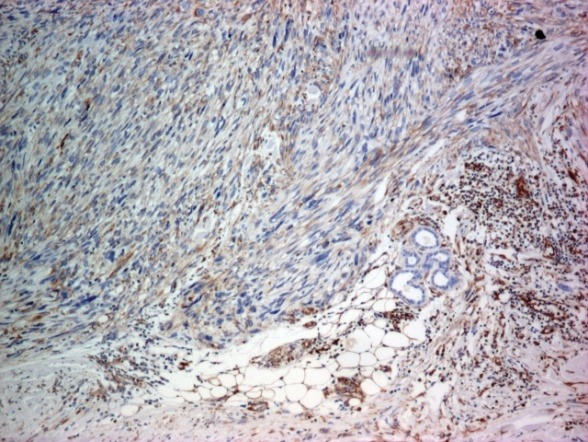
Immunohistochemical stain for vimentin: diffuse positive in tumor cells; hypodermis invasion (x 40)

**Fig. 5:**
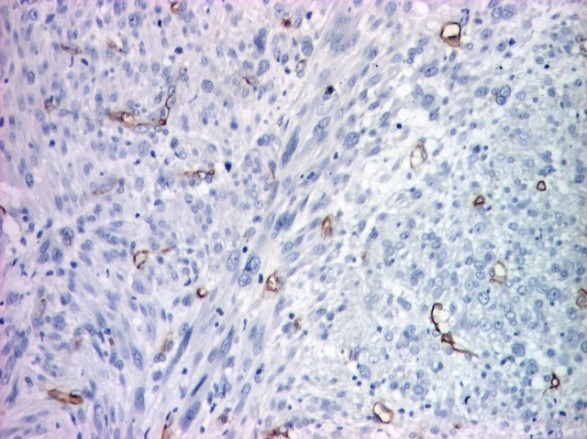
Immunohistochemical stain for ki 67: positive in 50% of tumor cells (x 40)

**Fig. 6:**
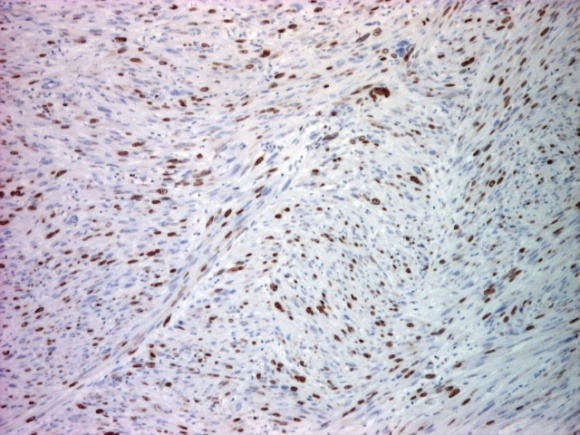
Immunohistochemical stain for CD34: negative in tumor and positive in vessels (x 100)

 Ki 67 was positive in 50% of tumor cells (**Fig. 6**). Based on the histopathological and immunohistochemical findings, the diagnosis of a cutaneous leiomyosarcoma was established. Staging investigations including computed tomography scans of the chest and abdomen were all negative. A complete resection with wide surgical margins had been performed. The patient was being followed up and was well without signs of disease three years after the resection of the tumor. 

## Discussion

Leiomyosarcoma is a rare neoplastic disease that was initially described by Montgomery and Winkelman in 1959 [**5**]. There are studies whose conclusions showed that this tumor develops most commonly in the lower limbs [**4**] but can occur on the face or trunk. Superficial leiomyosarcoma originates in the superficial dermis or subcutaneous tissue and represents about 3% of the soft tissue sarcomas. Different origins of these two entities gives them different clinical appearance and prognosis [**6**]. When the tumor is located in the dermis, its origin lays in the pilar follicle erector muscles or smooth muscles surrounding the sweat glands. If the tumor affects the subcutaneous tissue, its origin is in the smooth muscle of arteries and veins, in the scrotum muscles on the forehead, neck or trunk.

 The clinic of leiomyosarcoma consists in a firm dermal nodule, which can be painful, pruritic or paresthetic. The tumor is of 1-3 cm in diameter and can often be seen as a solitary formation. There may be multiple nodules evolving, located in hypodermis, in relation to the muscles of origin. Overlying skin is normal or has a red color. Rarely, due to skin trauma it may ulcerate. The clinical appearance of the subcutaneous location may be that of an indurated plaque. It can occur at any age, according to some authors, more frequently in the sixth or seventh decade of life. Both sexes are equally interested.

 Clinical diagnosis of cutaneous leiomyosarcoma is difficult, it can be determined only after histopathological examination. Differential diagnosis can be made with any solitary nodule developed on the skin or subcutaneous: lipoma, dermatofibroma, dermatofibrosarcoma, neurofibroma [**7**].

 Regarding the histological examination, the pathologist describes, in cutaneous leiomyosarcoma, a multitude of elongated smooth muscle cells, grouped in irregular bundles intersected with much richer than leiomyoma cellularity, a good differential diagnostic feature. The tumor is loosely bounded; cancer cells appear in the periphery of the tumor through the surrounding dermal collagen bundles. Constituent muscle cells are either well differentiated to poorly differentiated. The well-differentiated are elongated or spindle and color highlights show smooth, delicate myofibrils in the cytoplasm. Their nuclei elongated, rounded at the ends, are intensely colored. Poorly differentiated cells are pleomorphic and contain no myofibrils. Nuclear atypia is common. Dividing cells are numerous, their abundance being an important criterion for assessing the malignancy. Histologic differential diagnosis is difficult, calling into question other spindle cell tumors. Here we include atypical fibroxanthoma, spindle cell melanoma and spindle cell squamous cell carcinoma [**8,9**].

 Immunohistochemical examination brings evidences to support the diagnosis: the tumor expresses vimentin and smooth muscle actin. Vimentin was moderately positive; smooth muscle actin was intensely positive. The second marker is considered by some authors the most sensitive, it indicates smooth muscle differentiation [**10**] and has been described in some studies to be 100% positive [11,12]. The immunohistochemical markers represented by desmin, S-100 and CK were reported with a lower sensitivity. We have proceeded on diagnosis using the ki 67 marker, positive cell proliferation marker in about 50% of tumor cells. The CD34 marker was also positive in vessels but negative in the tumor.

 Regarding the evolution and prognosis, it is believed that approximately 50-60% of the cases recur after excision [**13**]. Recurrences have been reported even after the excision of tumors of small dimensions, of about 5 mm [**14**].

 Different authors have announced different rates of metastasis [**15-17**]. In 30-60% of the patients diagnosed with subcutaneous leiomyosarcoma and approximately 5% of the patients with cutaneous leiomyosarcoma, the disease complicated with metastases. Metastases are usually hematogenous. Lung is considered as the organ most frequently affected. The occurrence of metastases was noted in 1 to 3 years after the diagnosis of the primary tumor. Lymph node metastases are usually uncommon; they were reported in small percentage.

 Several factors are correlated to prognosis. These include tumor size, high mitotic rate, presence or absence of necrosis, intratumoral vascular invasion [**18,19**]. The survival rate for tumors smaller than 2 cm was of 95%, while in tumors that exceeded 5 cm, survival drops to 30% [**20**]. Prognosis often remains poor, despite a claim of radical therapies.

 Classic therapy of superficial leiomyosarcoma is represented by a wide excision with safety margin of 3 to 5 cm and re-excision in case of recurrence. When the tumor developed acral, complete excision may require amputation. The benefit of other therapeutic methods, such as chemotherapy and radiotherapy is contested, especially as radiotherapy is considered a causal factor. In the case of our patient, we performed a surgical excision with margins of at least 2 cm up to the fascia. The Mohs surgery would bring greater therapeutic benefits.

## Conclusions

Leiomyosarcoma is a rare entity whose clinical presentation may appear nonspecific, making the diagnosis difficult. Primary tumor excision with wide oncological safety margins is considered, when suitable case, the most appropriate method. Other therapeutic methods, such as radio- or chemotherapy are described as without significant benefits. Despite the claims of radical surgical treatment, due to recurrence rates, the prognosis remains poor. We recommend a long-term follow-up of patients to capture a subsequent malignant disease progression.
